# Association of person-centred maternity care during childbirth and future reproductive and health-seeking intentions among adolescents in Lira, Northern Uganda: a cross-sectional survey

**DOI:** 10.1080/16549716.2026.2721157

**Published:** 2026-08-25

**Authors:** Samson Udho, Sheila Elizabeth Clow

**Affiliations:** a Division of Nursing and Midwifery, Department of Health and Rehabilitation Sciences, Faculty of Health Sciences, University of Cape Town, Rondebosch, South Africa; b Department of Midwifery, Faculty of Nursing and Midwifery, Lira University, Lira, Uganda

**Keywords:** “Person-centred maternity care”, reproductive, health-seeking, intentions, adolescents

## Abstract

**Background:**

Person-centred maternity care (PCMC) is essential for delivering respectful, responsive, and high-quality care during childbirth. However, limited evidence exists on the association between PCMC during childbirth and adolescents’ future reproductive and health-seeking intentions.

**Objective:**

To examine the association between PCMC during childbirth and future reproductive and health-seeking intentions among adolescents in Lira, Northern Uganda.

**Methods:**

We conducted a community-based cross-sectional study among 560 adolescents aged 14–19 years who had given birth in public primary health facilities between March 2023 and July 2024. PCMC was measured using the validated PCMC scale, while future reproductive and health-seeking intentions were assessed using items adapted from the Community Survey Tool. Data were analysed using descriptive statistics and multivariable logistic regression, adjusting for socio-demographic and obstetric factors.

**Results:**

The median age of participants was 18 years (IQR: 18–19). Most adolescents intended to have another child (82%), return to the same facility for future childbirth (83%), and recommend the facility to others (85%). Overall, 77.7% reported moderate levels of PCMC. Adolescents reporting moderate and high PCMC were more likely to intend to return to the same facility for future childbirth (AOR = 2.84, 95% CI: 1.61–5.00; AOR = 5.60, 95% CI: 1.19–26.43, respectively). Adolescents reporting moderate PCMC were also more likely to recommend the facility to others (AOR = 4.31, 95% CI: 2.46–7.54).

**Conclusions:**

Higher levels of PCMC were associated with positive future health-seeking intentions among adolescents. Strengthening adolescent-responsive PCMC may enhance future engagement with skilled childbirth services and contribute to improved maternal health outcomes in low-resource settings.

## Paper Context

- **Main findings**: Adolescents who reported higher levels of person-centred maternity care during childbirth were more likely to intend to return to the same health facility for future childbirth and recommend the facility to others, although no association was observed with future fertility intentions.
- **Added knowledge**: This study adds important evidence from a low-resource setting by demonstrating that adolescents’ childbirth experiences can shape future health-seeking intentions, an area that remains underexplored in maternal health research.
- **Global health impact for policy and action**: The findings highlight the need for policies and interventions that strengthen respectful, responsive, and adolescent-centred maternity care to promote continued utilization of skilled childbirth services and improve maternal and newborn health outcomes.

## Background

Maternal mortality remains a significant global public health challenge, with over 700 women succumbing each day to preventable causes associated with pregnancy and childbirth [1]. Despite substantial progress evidenced by a decline of approximately 40% in maternal mortality since 2000, more than 90% of these fatalities occur in low- and lower-middle-income countries [2,3]. Consequently, enhancing the quality of care during childbirth is imperative for mitigating maternal and newborn morbidity and mortality rates. In addition to clinical effectiveness, there is a growing acknowledgment that women’s experiences of care encompassing treatment, communication, and support are integral to achieving favorable health outcomes and promoting sustained utilization of maternal health services [4–7].

The World Health Organization (WHO) Quality of Care Framework for Maternal and Newborn Health identifies both the provision of care and the experience of care as essential components of high-quality maternity services, emphasizing effective communication, respect and dignity, and emotional support for women and newborns in health facilities [5,7]. Building on this experiential dimension of quality care, person-centred maternity care (PCMC) has emerged as a pivotal framework for assessing and promoting women’s experiences during childbirth [5,7,8]. PCMC encompasses key elements of dignity and respect, effective communication, autonomy, and supportive care, while emphasizing responsiveness to women’s preferences, needs, and values throughout the childbirth process [5,7,8]. Evidence from diverse contexts indicates that higher levels of PCMC are associated with greater satisfaction with care, increased utilization of skilled birth services, and improved postpartum well-being [9]. Notably, positive childbirth experiences can significantly influence women’s future reproductive and health-seeking intentions, including decisions about having future births, returning to the same facility for childbirth, and recommending health facilities to others [10–13].

Future reproductive and health-seeking intentions refer to an individual or couple’s plans, desires, or intentions regarding having children in the future and utilization of maternity services [14]. It is a complex concept that entails intentions to give birth in the future, intentions to give birth in the same facility in the future, and intentions to recommend the same facility to a sister or a friend following previous childbirth experiences [8,15]. According to the theory of planned behavior by Ajzen [16], behaviors are influenced by intentions, which are determined by multiple prevailing factors. Childbearing decision-making is a complex process influenced by social, economic, political, individual, and health systems factors [17–19].

Despite the increasing emphasis on PCMC, adolescents remain an inadequately studied and particularly vulnerable and marginalized demographic within maternal health research [20]. This population often confronts compounded social, economic, and health system barriers, with their experiences of childbirth care frequently marred by stigma, discrimination, and restricted autonomy [21–25]. Such experiences may have enduring implications, not only affecting immediate satisfaction with care but also shaping future reproductive intentions and engagement with maternal health services. Nevertheless, empirical evidence examining how adolescents’ experiences of PCMC influence their future reproductive and health-seeking intentions particularly in low-resource settings remains scant.

In Uganda, particularly in the northern region, adolescents encounter high rates of early pregnancy and are often reliant on under-resourced public health facilities for childbirth [26–28]. Although initiatives to promote respectful maternity care and universal health coverage have increased [29,30], there is limited understanding of how adolescents perceive the quality of care received and how these perceptions impact their future reproductive and health-seeking intentions. Existing studies in Uganda and similar contexts have predominantly focused on adult women, leaving significant gaps in comprehending the unique needs and experiences of adolescent mothers [20].

To address these gaps, this study examined the association between PCMC and future reproductive and health-seeking intentions among adolescents in Lira, Northern Uganda, while adjusting for socio-demographic and obstetric factors. By concentrating on adolescents, a population at increased risk of adverse maternal outcomes and negative care experiences [21–25], this study aimed to provide evidence that informs the design of adolescent-responsive maternity services and strengthens maternal health systems in low-resource settings.

## Methods

### Study design

We conducted a community-based cross-sectional study to examine the association between PCMC and future reproductive and health-seeking intentions among adolescents in Lira, Northern Uganda. A cross-sectional design was appropriate as it enabled the simultaneous assessment of adolescents’ reported childbirth experiences (exposure) and their subsequent reproductive and health-seeking intentions (outcomes) within a defined population [31]. Data collection was carried out between March 2023 and July 2024. The study is reported in accordance with the Strengthening the Reporting of Observational Studies in Epidemiology (STROBE) guidelines [32]. This paper is part of a broader mixed-methods doctoral study that investigated adolescents’ experiences of facility-based childbirth in rural northern Uganda [33].

### Study setting

This study was conducted in Lira District, located approximately 342 km north of Kampala, the capital of Uganda. The district covers an area of 1,326 km^2^, of which 1,286 km^2^ is land, with the remainder consisting of water bodies and wetlands [34]. Administratively, Lira District is divided into two counties, Erute North and Erute South which are geographically separated by Lira City [35]. Erute North comprises the sub-counties of Aromo, Agweng, and Ogur, while Erute South includes Barr, Agali, and Amach [35].

The study was carried out in communities served by public primary health facilities (Health Centre III and Health Centre IV) within these sub-counties, where participating adolescents had delivered. Health Centre IIIs provide antenatal, intrapartum, and postnatal care, as well as Basic Emergency Obstetric and Newborn Care (BEmONC) services [36]. Health Centre IVs offer similar routine services but are additionally equipped to deliver Comprehensive Emergency Obstetric and Newborn Care (CEmONC), including advanced obstetric interventions [36].

Lira District was purposively selected due to its high burden of adolescent pregnancy and its predominantly rural, resource-constrained health system context [26–28]. Adolescents in this setting frequently rely on public primary health facilities for childbirth, where variations in the quality and experience of care are likely to influence subsequent health-seeking behaviours [37]. This makes the district a relevant setting for examining the relationship between person-centred maternity care and future reproductive and health-seeking intentions in a low-resource context.

### Study population and eligibility

The study focused on female adolescents aged 14–19 who had given birth within the two to six weeks prior to data collection at public health facilities in Lira District that offer maternity services. Participants were required to be able to communicate in either *Lango*, the local language, or English. Those excluded from the study included individuals who were unwell, unavailable at home, or occupied with other responsibilities during the data collection visits. Additional exclusions were applied to individuals employed by the selected health facilities, first-degree relatives (such as mothers, sisters, or cousins) of facility staff, and those who screened positive for probable postnatal depression using the three-item Edinburgh Postnatal Depression Scale [38]. This was done to prevent response bias that could result from severe psychological distress affecting their reporting.

### Sample size and sampling procedures

The sample size was calculated using the Kish–Leslie formula, with a target of 610 adolescents. This estimate was based on a 95% confidence level, a 5% margin of error, and an estimated prevalence of 60.3% for skilled attendance at childbirth among adolescents in Uganda [39]. We also included a 10% allowance for non-responses and applied a design effect of 1.5 to account for clustering.

To recruit study participants, we used a census sampling approach [40]. Given the limited number of adolescents eligible for facility-based childbirth in the district, we included all qualified participants to ensure a sufficient sample size and enhance the robustness of our findings. This method was considered appropriate and feasible due to the restricted and accessible target population during the study period. Consequently, we enrolled all adolescents who met the inclusion criteria from the district’s public health facilities until the desired sample size was reached.

### Recruitment of study participants

After obtaining all necessary ethical and administrative approvals, we began sharing information about the study in postnatal wards and clinics through posters. During these sessions, research assistants introduced themselves to potential participants. Using a standardized screening tool (Supplementary File 1), the research assistants assessed adolescents interested in learning more about the study. Those who met the eligibility criteria were invited to participate voluntarily. For those who consented, their contact details were recorded for follow-up interviews to be conducted in the community, two to six weeks after childbirth, at a convenient time. This timing was intentional, allowing participants ample opportunity to reflect on their childbirth experiences and ensuring that interviews took place outside the facility where they received care [41].

### Study variables

The outcome variable was future reproductive and health-seeking intentions (intentions to give birth, give birth in the same facility, and recommend the same facility to a sister or friend) following facility-based childbirth. The primary independent variable was the status of PCMC during childbirth. Other independent variables included socio-demographics (age, marital status, level of education, employment status, and socioeconomic status) and obstetric history (gravidity, parity, number of ANC visits, place of birth, mode & time of birth, number of babies, sex of baby, companionship during birth, complications during birth, newborn outcome).

### Data collection

Data were collected using a face-to-face survey using a paper-based structured questionnaire with closed-ended questions (Supplementary File 2). The questionnaire consisted of three sections, namely, socio-demographic characteristics, obstetric history, future reproductive and health-seeking intentions following facility-based childbirth. Future reproductive and health-seeking intentions were assessed using three items on the community survey tool (CST) [15]. The CST is a valid and reliable tool for measuring future reproductive and health-seeking intentions in low-income settings like Uganda [15]. Participants were asked if they had intentions to give birth in the future after their latest childbirth, use the same health facility for future childbirth, and recommend the same health facility to a sister or friend. Future fertility intention was recorded as ‘No’, ‘Yes’, or ‘Don’t Know’. Future intention to give birth in the same facility was as recorded as ‘Same health facility’, Different health facility”, ‘At my home’, ‘At someone else’s home’, or ‘Don’t Know’. Meanwhile, future intention to recommend the same facility to a sister or friend was measured on a 5-point Likert scale (0 = strongly disagree, 1 = Agree, 2 = Neutral, 3 = Disagree, and 4 = strongly disagree) based on response to the statement, ‘I would recommend this health facility to a sister or friend’.

PCMC during childbirth was assessed using the PCMC scale [42]. The reliability test of the tool in this study using Cronbach’s Alpha showed that the overall PCMC scale was reliable for this population (*r* = 0.86) as well as the sub-scales of dignity and respect (*r* = 0.74), supportive care (*r* = 0.78), and communication and autonomy (*r* = 0.80). The overall PCMC scale is a 30-item scale with a total score range of 0 to 90 where each item has a 4-point response scale, i.e. 0 (‘no, never’), 1(‘yes, a few times’), 2 (‘yes, most of the time’) and 3 (‘yes, all the time’) [42]. The overall PCMC and sub-scale scores were categorised into ‘low, moderate, and high’ [42].

Data were collected by three trained research assistants. The team comprised a social worker, a counselor, and a youth worker, each contributing relevant expertise in engaging adolescents. The research assistants conducted interviews in participants’ preferred language, either *Lango* (the local dialect) or English. Each survey session lasted approximately 30 minutes.

### Data analysis

At the close of each data collection day, completed questionnaires were reviewed to ensure all items had been filled in appropriately. A data entry template was developed in STATA version 17 [43], incorporating built-in accuracy checks to minimize entry errors. Prior to analysis, the dataset was examined for missing values, inconsistencies, and out-of-range responses. We applied both descriptive and inferential statistical analyses. Continuous variables were tested for normality using the Shapiro–Wilk test. Normally distributed variables were summarized using means and standard deviations/interquartile ranges, while non-normally distributed variables were summarized using medians and interquartile ranges.

A bivariate and multivariate logistic regression was performed to test the association between the PCMC and adolescents’ future reproductive and health-seeking intentions while controlling for the influence of socio-demographic and obstetric characteristics. Variables with a *p*-value < 0.05 in the bivariate analyses were included in the multivariate model. The analysis was conducted at a 95% level of significance, and the corresponding adjusted odds ratio (AOR) was reported. All the variables in the model were assessed for multi-collinearity using the VIF and variables with VIF > 10 were removed from the model [44].

## Results

### Study participants

**Figure 1.**
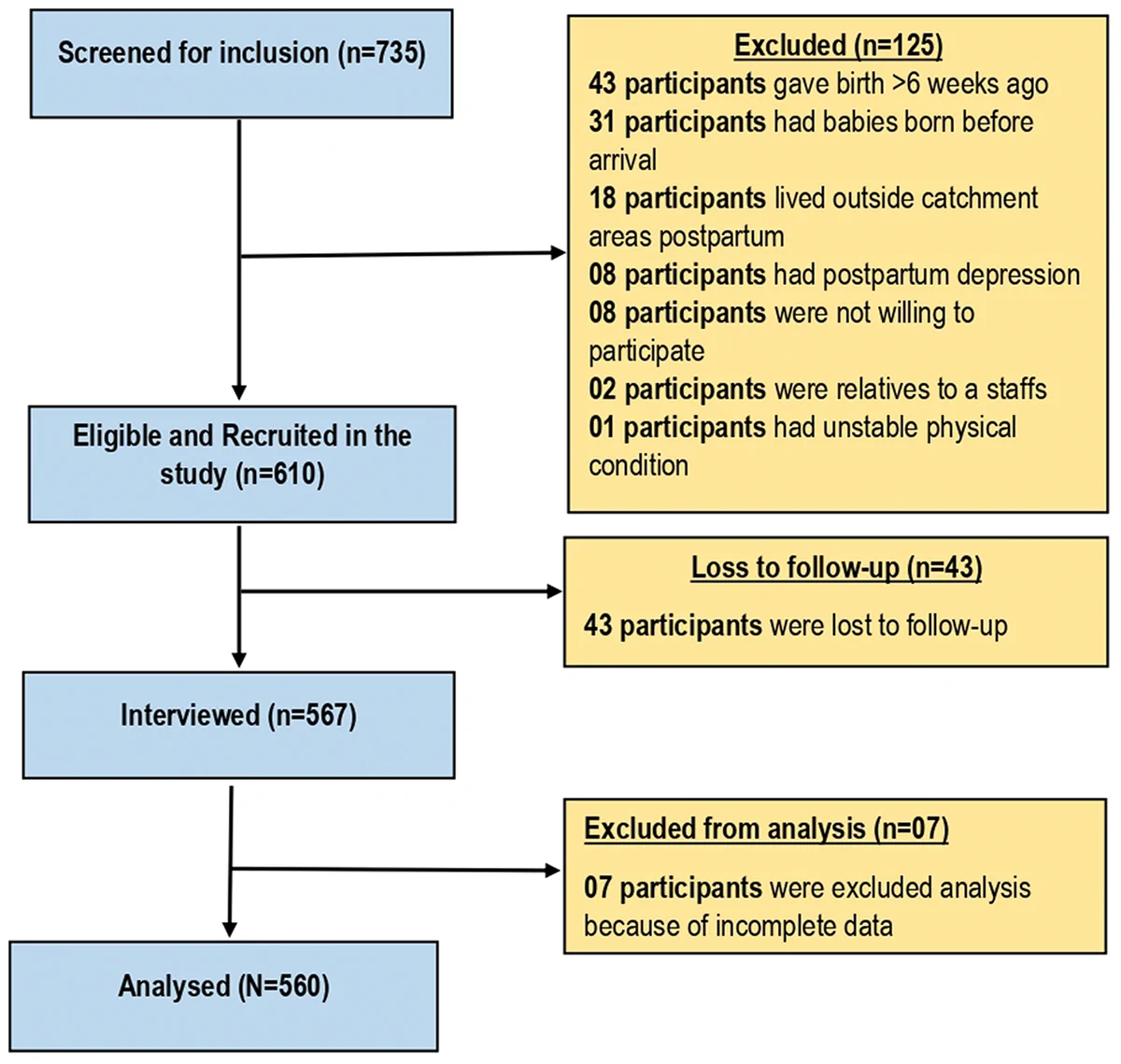
Flowchart for recruitment of study participants.

A total of 735 adolescents were screened for eligibility, of whom 610 met the inclusion criteria and were enrolled in the study. Among those enrolled, 567 completed the survey, yielding a response rate of 92.95% (567/610). Seven participants were excluded from the analysis due to incomplete data, resulting in a final analytical sample of 560. Details of the screening process, exclusions, and final sample are presented in Figure 1.

### Sociodemographic characteristics of study participants

The participants had a median age of 18 years (IQR: 18–19). Nearly all had some formal education (97.7%), though most were no longer attending school (96.4%). The majority were married (85.0%), engaged in agricultural work (76.8%), and belonged to the middle wealth category (61.3%) (Table 1).

**Table 1.** Sociodemographic characteristics of study participants (*N* = 560).

Characteristics	n (%)	Would like to have another child (n = 463) COR [95% CI]	Same facility next childbirth (n = 460) COR [95% CI]	Would recommend the facility (n = 474) COR [95% CI]
**Age**				
14–17	92 (16.4)	Ref.	Ref.	Ref.
18–19	468 (83.6)	1.96 [1.16, 3.29]*	1.20 [0.68, 2.12]	1.69 [0.97, 2.96]
**School attendance**				
No	540 (96.4)	Ref.	Ref.	Ref.
Yes	20 (3.6)	1.24 [0.36, 4.32]	–	3.55 [0.57, 26.87]
**Level of education**				
No formal education	13 (2.3)	Ref.	Ref.	Ref.
Primary education	509 (90.9)	1.32 [0.36, 4.91]	1.50 [0.40, 5.55]	1.61 [0.43, 5.97]
Secondary education	38 (6.8)	3.50 [0.61, 20.10]	0.79 [0.22, 4.30]	3.50 [0.61, 20.10]
**Occupation**				
Agricultural labour	430 (76.8)	Ref.	Ref.	Ref.
Casual labour	59 (10.5)	0.72 [0.37, 1.41]	0.88 [0.41, 1.89]	1.28 [0.55, 2.93]
Self-employed	37 (6.6)	0.74 [0.33, 1.69]	0.21 [0.10, 0.42]**	0.53 [0.24, 1.19]
Unemployed	34 (6.1)	0.95 [0.38, 2.39]	0.26 [0.12, 0.54]**	0.66 [0.28, 1.59]
**Marital status**				
Single	24 (4.3)	Ref.	Ref.	Ref.
Married	476 (85.0)	3.11 [1.31, 7.36]*	3.26 [1.37, 7.72]*	2.65 [1.06, 6.64]*
Cohabiting	48 (8.6)	2.60 [0.87, 7.80]	2.02 [0.70, 5.86]	1.11 [0.37, 3.29]
Divorced/separated	12 (2.1)	0.84 [0.20, 3.46]	1.80 [0.38, 8.45]	2.06 [0.36, 11.91]
**Wealth quantile**				
Lowest quantile	163 (29.1)	Ref.	Ref.	Ref.
Middle quantile	343 (61.3)	1.11 [0.69, 1.79]	1.27 [0.79, 2.05]	1.66 [1.02, 2.70]*
Highest quantile	54 (9.6)	1.17 [0.52, 2.65]	1.70 [0.71, 4.11]	2.56 [0.96, 6.98]

Note. *Significant Variable at *p* < 0.05. **Significant Variable at *p* < 0.001. Ref, Reference Category. COR, Crude Odds Ratio. CI, Confidence Interval.

### Obstetric characteristics of study participants

Most participants were primiparous (85.0%) and delivered in a Health Centre III facility (68.6%). The vast majority had a spontaneous vaginal birth (95.9%), experienced no maternal complications (62.0%), and reported no neonatal complications (96.3%) (Table 2).

**Table 2.** Obstetric characteristics of study participants (*N* = 560).

Characteristics	n (f)	Would like to have another child COR [95% CI]	Same facility next childbirth COR [95% CI]	Would recommend facility to sister or friends COR [95% CI]
**Gravidity**				
1	476 (85.0)	Ref.	Ref.	Ref.
>1	84 (15.0)	1.22 [0.65, 2.31]	1.66 [0.82, 3.33]	0.89 [0.48, 1.67]
**Parity**				
1	501 (89.5)	Ref.	Ref.	Ref.
>1	59 (10.5)	1.07 [0.52, 2.20]	1.63 [0.72, 3.70]	0.77 [0.38, 1.55]
**Antenatal Visits**				
≤4	195 (34.8)	Ref.	Ref.	Ref.
5–7	312 (55.7)	0.82 [0.52, 1.31]	0.76 [0.47, 1.23]	0.90 [0.54, 1.49]
≥8	53 (9.5)	1.96 [0.72, 5.29]	1.19 [0.49, 2.90]	0.82 [0.36, 1.86]
**Level of Health Facility**				
HC IV	176 (31.4)	Ref.	Ref.	Ref.
HC III	384 (68.6)	0.87 [0.54, 1.40]	2.08 [1.33, 3.26]*	3.04 [1.90, 4.87]**
**Mode of Childbirth**				
Vaginal birth	537 (95.9)	Ref.	Ref.	Ref.
Caesarean section	23 (4.1)	2.34 [0.54, 10.16]	0.46 [0.18, 1.15]	0.86 [0.28, 2.58]
**Episiotomy [*n* = 537]**				
No	446 (83.0)	Ref.	Ref.	Ref.
Yes	91 (17.0)	0.25 [0.15, 0.41]**	0.72 [0.41, 1.26]	0.99 [0.53, 1.85]
**Time of Childbirth**				
Day	148 (26.4)	Ref.	Ref.	Ref.
Evening	182 (32.5)	1.11 [0.61, 2.02]	3.04 [1.65, 5.60]**	2.46 [1.33, 4.54]*
Night	230 (41.1)	0.71 [0.42, 1.23]	1.50 [0.90, 2.46]	1.65 [0.97, 2.81]*
**Birth Companion**				
No	178 (31.8)	Ref.	Ref.	Ref.
Yes	382 (68.2)	2.23 [1.43, 3.46]**	0.34 [0.19, 0.60]**	0.21 [0.10, 0.43]**
**Maternal Complications**				
No	350 (62.5)	Ref.	Ref.	Ref.
Yes	210 (37.5)	0.34 [0.22, 0.53]**	0.92 [0.59, 1.44]	1.14 [0.71, 1.84]
**Sex of Baby**				
Male	268 (47.9)	Ref.	Ref.	Ref.
Female	292 (52.1)	0.83 [1.54, 1.28]	1.26 [0.81, 1.95]	1.38 [0.87, 2.19]
**Newborn Complications**				
No	539 (96.3)	Ref.	Ref.	Ref.
Yes	21 (3.7)	0.92 [0.30, 2.80]	0.89 [0.29, 2.69]	0.76 [0.25, 2.32]
**Status of Newborn**				
Dead	13 (2.3)	Ref.	Ref.	Ref.
Alive	547 (97.7)	0.83 [0.18, 3.82]	2.17 [0.65, 7.19]	1.68 [0.45, 6.22]

Note. *Significant Variable at *p* < 0.05. **Significant Variable at *p* < 0.001. Ref, Reference Category. COR, Crude Odds Ratio. CI, Confidence Interval.

### Future reproductive and health-seeking intentions following childbirth

More than three-quarters of the participants would like to have another child in the future (82.14%), would return to the same health facility for the next childbirth (82.68%), and agree that they would recommend where they gave birth to a sister or a friend (84.65%) (Table 3).

**Table 3.** Future reproductive and health-seeking intentions of study participants (*N* = 560).

Future Reproductive and Health-Seeking Intentions	Frequency (n)	Percent (%)
**Would like to have another child**		
No	14	2.50
Yes	460	82.14
Don’t Know	86	15.36
**Choice for next childbirth**		
Same health facility	463	82.68
Different health facility	20	3.57
At home	00	00.00
Don’t know	77	13.75
**I would recommend the same facility to a sister or friend**		
Strongly agree	107	19.11
Agree	367	65.54
Neutral	75	13.39
Disagree	8	1.43
Strongly disagree	3	0.54

## Adolescents’ perceptions of person-centred maternity care during childbirth

Overall, more than three-quarters of the participants (77.74%) perceived the person-centred maternity care (PCMC) they received during childbirth as moderate. Likewise, the majority reported moderate levels of dignity and respect (75.09%) and supportive care (78.00%). In contrast, fewer than half of the participants (45.89%) perceived communication and autonomy during childbirth as moderate (Table 4).

**Table 4.** Adolescents’ perceptions of person-centred maternity care during childbirth among study participants (*N* = 560).

PCMC Sub-scale	n (%)	Would like to have another child COR [95% CI]	Same facility next childbirth COR [95% CI]	Would recommend facility to sister or friends COR [95% CI]
**PCMC**				
Low	88 (15.80)	Ref.	Ref.	Ref.
Moderate	433 (77.74)	0.82 [0.44, 1.53]	3.36 [2.02, 5.59]**	5.69 [3.39, 9.54]**
High	36 (6.46)	1.17 [0.39, 3.54]	9.71 [2.19, 43.14]*	–
**Dignity and Respect**				
Low	80 (14.34)	Ref.	Ref.	Ref.
Moderate	419 (75.09)	0.67 [0.33, 1.35]	0.99 [0.54, 1.83]	1.85 [1.04, 3.28]*
High	59 (10.57)	0.38 [0.16,0.92]*	4.31 [1.19, 15.65]*	9.50 [2.12, 42.49]*
**Communication and Autonomy**				
Low	187 (33.39)	Ref.	Ref.	Ref.
Moderate	257 (45.89)	0.74 [0.45, 1.22]	5.05 [2.98, 8.53]**	3.97 [2.36, 6.66]**
High	116 (20.71)	0.88 [0.47, 1.64]	4.30 [2.20, 8.41]**	9.49 [3.67, 24.51]**
**Supportive Care**				
Low	61 (10.91)	Ref.	Ref.	Ref.
Moderate	436 (78.00)	0.43 [0.18, 1.02]	2.39 [1.32, 4.31]*	5.64 [3.17, 10.03]**
High	62 (11.09)	1.24 [0.36, 4.31]	9.59 [2.67, 34.41]*	–

*Significant variable at *p* < 0.05. *Significant variable at *p* < 0.001. Ref, Reference category. (-), Missing or outrageous values.

## Multivariate analysis examining the association between adolescents’ perceptions of person-centred maternity care during childbirth and future reproductive and health-seeking intentions of study participants (*N* = 560)

The multivariate logistic regression in model 1 (*with the PCMC as the main independent variable while controlling for other socio-demographic and obstetric variables*) showed that there was no evidence of an association between perceived moderate and high levels of PCMC during childbirth and adolescents’ intention to have another child in the future (AOR = 0.90, 95% CI [0.42, 1.94] and AOR = 1.59, 95% CI [0.47, 5.37] respectively). Other variables independently associated with intentions to have another child in the future were being married (AOR = 4.49, 95% CI [1.53, 13.22]), 5–7 antenatal visits (AOR = 0.56, 95% CI [0.32, 0.98]), episiotomy (AOR = 0.34, 95% CI [0.19, 0.59]), and maternal complication (AOR = 0.44 [0.24, 0.79]).

Participants who had perceptions of moderate and high levels of PCMC during childbirth had 2.84 and 5.60 higher odds, respectively, of choosing the same facility for the next childbirth compared to those who had perceptions of low PCMC levels (AOR = 2.84, 95% CI [1.61, 5.00] and AOR = 5.60, 95% CI [1.19, 26.43] respectively). Other variables independently associated with intentions to choose the same facility for the next childbirth were being self-employed (AOR = 0.26 [0.11, 0.59]) or unemployed/homemaker (AOR = 0.37 [0.15, 0.91]) and giving birth in the evening hours (AOR = 2.53 [1.34, 4.76]).

Participants who had perceptions of moderate levels of PCMC during childbirth had 4.31 higher odds of recommending the same facility to a sister or a friend compared to those who had perceptions of low levels (AOR = 4.31, 95% CI [2.46, 7.54]). The only other variable that was independently associated with intentions to recommend the same facility to a sister or a friend was giving birth in the evening hours (AOR = 2.01 [1.03, 3.92]) (Table 5).

**Table 5.** Association between adolescents’ perceptions of PCMC during childbirth and future reproductive and health-seeking intentions among study participants (*N* = 560).

Characteristics	Adolescents’ Future Reproductive and Health-Seeking Intentions		
	Would like to have another child (*n* = 463) AOR [95% CI]	Same facility next childbirth (*n* = 460) AOR [95% CI]	Would recommend facility to sister or friends (*n* = 474) AOR [95% CI]
**PCMC**			
Low	Ref.	Ref.	Ref.
Moderate	0.90 [0.42, 1.94]	2.84 [1.61, 5.00]**	4.31 [2.46, 7.54]**
High	1.59 [0.47, 5.37]	5.60 [1.19, 26.43]*	–
**Age**		NA	
14–17	Ref.		Ref.
18–19	1.99 [1.12, 3.56]*		1.21 [0.60, 2.44]
**Level of education**			
No formal education	Ref.	Ref.	Ref.
Primary education	1.13 [0.24, 5.29]	–	1.02 [0.20, 5.33]
Secondary education	4.40 [0.54, 36.03]	–	3.03 [0.38, 24.42]
**Occupation**			
Agricultural labour	Ref.	Ref.	Ref.
Casual labour	–	0.85 [0.35, 2.07]	0.93 [0.34, 2.57]
Self-employed	–	0.26 [0.11, 0.59]*	0.67 [0.25,1.80]
Unemployed/homemaker	–	0.37 [0.15, 0.91]*	1.04 [0.34, 3.18]
**Marital status**			
Single	Ref.	Ref.	Ref.
Married	4.49 [1.53, 13.22]*	1.90 [0.66, 5.36]	1.17 [0.35, 3.94]
Cohabiting	3.47 [0.91,13.28]	1.70 [0.50, 5.73]	0.93 [0.26, 3.39]
Divorced/separated	0.62 [0.11, 3.58]	1.41 [0.26, 7.61]	1.60 [0.25, 10.06]
**Wealth quantile**			
Lowest quantile	Ref.	Ref.	Ref.
Middle quantile	–	–	1.53 [0.89, 2.64]
Highest quantile	–	–	2.14 [0.64, 7.13]
**Number of Antenatal Visits**			
≤4	Ref.	Ref.	Ref.
5–7	0.56 [0.32, 0.98]*	–	–
≥8	1.03 [0.34, 3.10]	–	–
**Health Facility Level**			
HC IV	Ref.	Ref.	Ref.
HC III	–	1.20 [0.68, 2.12]	1.78 [0.97, 3.27]
**Episiotomy [*n* = 537]**			
No	Ref.	Ref.	Ref.
Yes	0.34 [0.19, 0.59]**	–	–
**Time of Childbirth**			
Day	Ref.	Ref.	Ref.
Evening	–	2.53 [1.34, 4.76]*	2.01 [1.03, 3.92]*
Night	–	1.20 [0.68, 2.11]	1.32 [0.71, 2.44]
**Birth Companion**			
No	Ref.	Ref.	Ref.
Yes	1.76 [0.98, 3.16]	0.66 [0.34, 1.31]	0.47 [0.20, 1.11]
**Maternal Complications**			
No	Ref.	Ref.	Ref.
Yes	0.44 [0.24, 0.79]*	–	–

Note. *Significant Variable at *p* < 0.05. **Significant Variable at *p* < 0.001. Ref, Reference category. NA, Not Applicable. AOR, Adjusted Odds Ratio. CI, Confidence Interval. (-), Missing or Outrageous Values.

The multivariable logistic regression analysis (Model 2), which included the PCMC sub-scales as the primary independent variables while adjusting for socio-demographic and obstetric characteristics, showed that the inverse association observed in the unadjusted analysis between perceived dignity and respect during childbirth and adolescents’ intentions to have another child was no longer statistically significant. Specifically, compared with adolescents who reported low levels of dignity and respect, those reporting moderate (AOR = 0.86, 95% CI: 0.38–1.93) and high (AOR = 0.53, 95% CI: 0.18–1.53) levels had no significant differences in their intentions to have another child, indicating that dignity and respect was not an independent predictor of future childbearing intentions. Similarly, there was no evidence of an independent association between perceived dignity and respect during childbirth and adolescents’ intentions to return to the same facility for a future childbirth among those reporting moderate (AOR = 0.69, 95% CI: 0.35–1.38) or high (AOR = 1.26, 95% CI: 0.34–4.70) levels of dignity and respect. Likewise, perceived dignity and respect was not significantly associated with adolescents’ likelihood of recommending the facility to a sister or friend among those reporting moderate (AOR = 1.11, 95% CI: 0.57–2.13) or high (AOR = 2.16, 95% CI: 0.42–11.15) levels.

Participants who had perceptions of high levels of communication and autonomy during childbirth had 3.42 higher odds of recommending the same facility to a sister or a friend compared to those who had perceptions of low levels (AOR = 3.42 [1.14, 10.24]).

Adolescents who perceived high levels of supportive care during childbirth had 5.10 higher odds of choosing the same facility in the future compared to those who had low levels (AOR = 5.10, 95% CI [1.20, 21.59]). Those who had perceptions of moderate levels of supportive care had 4.52 higher odds of recommending the same facility to a sister or a friend compared to those who had low levels (AOR = 4.52 [2.36, 8.66]) (Table 6).

**Table 6.** Association between adolescents’ perceptions of PCMC sub-scales during childbirth and future reproductive and health-seeking intentions among study participants (*N* = 560).

PCMC Sub-scale^a^	Adolescents’ Future Reproductive and Health-Seeking Intentions		
	Would like to have another child (*n* = 463) AOR [95% CI]	Same facility next childbirth (*n* = 460) AOR [95% CI]	Would recommend facility to sister or friends (*n* = 474) AOR [95% CI]
**Dignity and Respect**			
Low	Ref.	Ref.	Ref.
Moderate	0.86 [0.38, 1.93]	0.69 [0.35, 1.38]	1.11 [0.57, 2.13]
High	0.53 [0.18, 1.53]	1.26 [0.34,4.70]	2.16 [0.42, 11.15]
**Communication and Autonomy**			
Low	Ref.	Ref.	Ref.
Moderate	1.10 [0.53,2.12]	1.75 [0.86, 3.53]	1.67 [0.90, 3.09]
High	1.03 [0.45, 2.36]	2.24 [0.97, 5.18]	3.42 [1.14, 10.24]*
**Supportive Care**			
Low	Ref.	Ref.	Ref.
Moderate	0.52 [0.17,1.63]	1.75 [0.86, 3.53]	4.52 [2.36, 8.66]**
High	1.31 [0.30,5.69]	5.10 [1.20, 21.59]*	–

Note. *Significant variable at *p* < 0.05. **Significant variable at *p* < 0.001. Ref, Reference category. (-), Missing or Outrageous Values.

^a^The model controlled for age, school attendance, level of education occupation, occupation, marital status, wealth quantile, parity, gravidity, place of childbirth, birth companionship, sex of baby, newborn complication, the status of the baby, number of antenatal care visit, mode of childbirth, episiotomy, time of childbirth, and maternal complications.

## Discussion

We examined the association between PCMC during childbirth and future reproductive and health-seeking intentions among adolescents in Lira, Northern Uganda. We found that although most adolescents reported moderate levels of PCMC, future reproductive and health-seeking intentions were high, with the majority expressing intentions to have another child, return to the same facility for future childbirth, and recommend the facility to others. These findings suggest that adolescents generally maintained confidence in maternity services despite experiencing only moderate levels of person-centred care. Importantly, higher levels of PCMC were associated with increased likelihood of intending to return to the same facility and recommending it to others, highlighting the critical role of positive childbirth experiences in shaping future health-seeking intentions. Our findings underscore the importance of strengthening adolescent-responsive, person-centred maternity care as a strategy for promoting continued utilization of skilled childbirth services and improving maternal health outcomes in low-resource settings.

This study found that 82% of the participants expressed a desire to have another child in the future, accounting for more than three-quarters of the participants. This result aligns with a Thai hospital-based study in which 87% of multiparous pregnant adolescents (mean age 18.09 ± 1.7 years) intended to have a repeat pregnancy during the index pregnancy [45]. Similar patterns have been documented in Eastern Uganda, where first-time adolescent mothers frequently intended subsequent births, often as part of completing their family size or in response to partner pressure, despite recognised risks of rapid repeat pregnancy [46]. The high prevalence of future fertility intentions observed in this study is plausible given that participants were between 14 and 19 years of age and that nearly nine in ten (89.5%) were first-time mothers. However, after adjusting for socio-demographic and obstetric characteristics, overall perceptions of PCMC were not significantly associated with adolescents’ intentions to have another child. Instead, future fertility intentions were independently associated with older adolescent age (18–19 years), being married, attending five to seven antenatal care visits, experiencing an episiotomy, and maternal complications. These findings suggest that adolescents’ reproductive intentions are influenced primarily by their demographic, social, and reproductive circumstances rather than by their perceptions of the quality of maternity care received during childbirth. This finding is consistent with evidence that fertility intentions among adolescents are shaped by broader sociocultural norms, marital expectations, partner influences, and desired family size, particularly in settings where childbearing remains highly valued [46,47]. Consequently, while improving PCMC remains essential for enhancing women’s childbirth experiences and promoting positive health-seeking behaviours, it may not substantially influence adolescents’ future fertility intentions, which appear to be driven by factors beyond the childbirth care experience.

Similarly, this study found that more than three-quarters of the participants intended to return to the same health facility for a future childbirth. This finding is consistent with a study conducted in Kenya, which reported that 85% of women would choose the same provider or health facility for their subsequent childbirth [12]. In the present study, the intention to return to the same facility should be interpreted within the broader context of healthcare access rather than as an indicator of the perceived quality of childbirth care. In Northern Uganda, adolescents often have limited access to alternative public health facilities, may reside considerable distances from the nearest facility, and frequently lack the financial resources to seek care in private facilities. Consequently, structural factors such as geographical accessibility, availability of services, and affordability are likely to play an important role in shaping future health facility choices [9,48–51]. These findings underscore the importance of addressing structural barriers to maternal healthcare access while continuing to improve the quality and PCMC in public health facilities.

Recommending a sister or a friend to access healthcare in the same facilities underscores the perception of quality of care. Like the preference to give birth in the same facility, mast of the participants (84.6%) agreed that they would recommend their birthplace to a sister or friend. This is likely because these participants had largely favourable childbirth experiences. These results align with a multi-country study conducted in Ghana, Guinea, Myanmar, and Nigeria, among 2672 women which revealed that 90% of these women would recommend their birthplace to others, such as friends or family [52]. Similar findings were also reported in studies conducted in Italy (79.3%), Mozambique (94.2%), and Guatemala (85%) [10,11,53]. The high level of agreement among women including adolescents in recommending their birthplace to others may reflect the degree to which care was perceived and their families’ preferences, needs, and values during pregnancy and childbirth. These findings underscore the importance of positive childbirth experiences in shaping individuals’ perceptions of care and health-seeking patterns.

One of the interesting findings of this study was that the overall PCMC or sub-domains were not associated with adolescents’ fertility intentions. This finding is consistent with a study conducted in Kenya which found no evidence of an association between PCMC scores and women’s fertility intentions [12]. However, this finding contradicts previous studies that found evidence of association between domains of PCMC such as supportive care, dignity, and respect and intentions to give birth in the future [10,11,13,52,54]. The differences in findings could be due to variations in study power and participant characteristics. For example, the study conducted by Preis, Tovim [13] among Israel’s women found that most of the participants (88%) had decided prenatally on their desired number of children which speaks to a clear fertility plan among those in that context.

It is also worth noting that adolescents are more likely to experience mistreatment during childbirth than older women [21–24], and this increases their chances of normalising disrespect and abuse during childbirth. This could potentially minimise the influence of disrespect and abuse on their decision-making regarding future childbearing. The discrepancies between the findings of this study and previous studies call for further research on the association between PCMC and women’s future reproductive and health-seeking intentions.

PCMC fosters an environment that promotes trust, satisfaction, and positive relationships between women and healthcare providers [8]. This study found that participants who had perceptions of moderate and high levels of PCMC during childbirth said that they would return to the same health facility for subsequent childbirth compared to those with low levels. This was particularly evident in adolescent participants who received appropriate supportive care, communication, and autonomy during childbirth. This finding aligns with the results of a study conducted in Kenya, Guatemala, and Italy which revealed that women with higher PCMC scores had significantly higher odds of expressing a willingness to return to the same facility for future childbirth compared to those with low PCMC scores [10–12]. Therefore, future interventions aimed at improving adolescents’ utilisation of maternal and newborn care services should prioritise strengthening the implementation of PCMC, particularly in terms of supportive care, communication between clients and providers, and promoting clients’ autonomy during care.

Participants who had perceptions of moderate and high levels of PCMC during childbirth said that they would recommend the facility to a sister or friend compared to those who had perceptions of low levels with PCMC sub-domains of supportive care, communication, and autonomy playing key roles in the observed association. This finding is plausible because implementing PCMC creates an environment where adolescents feel confident and connected to the facility, making them comfortable recommending it to others [8]. This result is consistent with the results of studies conducted in Kenya, Guatemala, Ghana, Guinea, Myanmar, and Nigeria, which also found that implementing PCMC is associated with women’s likelihood of recommending the facility [11,12,52]. These results underscore the importance of implementing PCMC, especially the aspects of dignity and respect, communication between clients and providers, and the promotion of clients’ autonomy in fostering confidence and connection to the facility.

### Strengths and limitations

This study focused on an understudied population, adolescents, and included a sufficiently large sample. It used the validated PCMC and CST instruments, which demonstrated strong validity and reliability in this population, thereby increasing confidence in the findings. Several limitations should be considered. First, the cross-sectional design precludes causal inference; therefore, the observed associations between PCMC and future reproductive and health-seeking intentions should be interpreted as correlational. Second, both PCMC and future reproductive and health-seeking intentions were assessed using self-reported measures, which may be subject to recall bias. This risk was mitigated by conducting interviews within 2–6 weeks postpartum, when participants’ recollection of childbirth experiences was likely to remain relatively accurate. Finally, the study was restricted to adolescents who gave birth in public primary health facilities in one region of Northern Uganda, which may limit generalizability to older women, private facilities, or other settings. Nonetheless, the study context shares many characteristics with other low-resource settings in sub-Saharan Africa, including constrained health-system resources and challenges in delivering person-centred maternity care, suggesting that the findings may have relevance for similar populations and contexts.

## Conclusion

Ensuring positive childbirth experiences for adolescents remains an important challenge for maternal health systems in low-resource settings. This study demonstrates that person-centred maternity care is associated with adolescents’ future reproductive and health-seeking intentions, particularly their willingness to return to the same facility for childbirth and recommend it to others. These findings reinforce the importance of the experience-of-care domain within the WHO Quality of Care Framework for Maternal and Newborn Health and suggest that respectful, responsive, and supportive maternity care can influence future engagement with skilled childbirth services. Strengthening adolescent-responsive person-centred maternity care should therefore be prioritized within quality improvement initiatives to promote continued utilization of maternal health services and contribute to improved maternal and newborn health outcomes. Further research is needed to explore the mechanisms through which specific dimensions of person-centred care, particularly dignity and respect, shape adolescents’ future reproductive and health-seeking decisions.

## Supplementary Material

STROBE Checklist.docx

Supplementary Material 2_Questionnaire.docx

Supplementary Material 1_Screening Tool.docx

## Data Availability

The datasets generated and analysed during the current study are not publicly available as they were collected for a PhD degree and no provisions were made to make them publicly available in terms of participants’ consent. However, the data is available from the corresponding author on reasonable request.
