# Supplementary material for: Association of person-centred maternity care during childbirth and future reproductive and health-seeking intentions among adolescents in Lira, Northern Uganda: a cross-sectional survey
Source: Glob Health Action. 2026 Aug 25;19(1):2721157. doi: 10.1080/16549716.2026.2721157 (PMC13520842; doi:10.1080/16549716.2026.2721157)
Supplement: Supplementary file 1 [file ZGHA_A_2721157_SM5954.docx]

**Supplementary Material 1: Screening Tool**

**Introduction**

I am Samson Udho, a nurse/midwife and currently a student at the University of Cape Town, South Africa where I’m doing research to get a Doctoral Degree. My study is being supervised by Professor Sheila E. Clow who works at the same University. We would like to find out what teenage mothers go through while giving birth in government health facilities. The information you will share may help us to understand what young women in your age group go through while giving birth in government maternity facilities so that we find ways to promote respectful maternity care for teenagers.

I will ask you six questions to check whether you are eligible to participate in the study. I will read the questions to you and you will respond to the questions with either a “no” or “yes” answer.

**Screening questions:**

1. Were you admitted to this health facility for childbirth?
0. No
1. Yes
2. Are you 14-19 years of age?
0. No
1. Yes
3. Are you a staff member of this health facility?
0. No
1. Yes
4. Do you have a first-degree relative (parent, guardian, brother, sister) in this health facility?
0. No
1. Yes
5. Will you live within Amach, Ogur, Barr, Barr, Agweng, and Aromo sub-county (Appendix I) at two to six weeks after childbirth?
0. No
1. Yes
6. Are you willing to participate in this study two to six weeks after childbirth?
0. No
1. Yes
7. Do you feel you are now in a stable health condition?
0. No
1. Yes
8. I have blamed myself unnecessarily when things went wrong.
0. No
1. Yes
9. I have felt scared or panicky for no very good reason.
0. No
1. Yes
10. I have been anxious or worried for no good reason.
0. No
1. Yes

**Note:** The woman is eligible to participate in the study if responses to question 1, 2, 5, & 6= ‘’Yes’’ and questions 3, 4, 7, 8, 9, and 10= ‘’No’’

Is the woman eligible?

0. Not eligible
1. Eligible

If the woman is “Not eligible”, sign, date, and do not recruit the woman into the study.

Sign: ……………………………………………. Date: ……………………………………

If the woman is “Eligible”, sign, date, and recruit the woman into the study

Sign: ……………………………………………………. Date: ………………………………

Recruitment number: ……………….Telephone: …………… Address: ………………………
