# Supplementary material for: Association of person-centred maternity care during childbirth and future reproductive and health-seeking intentions among adolescents in Lira, Northern Uganda: a cross-sectional survey
Source: Glob Health Action. 2026 Aug 25;19(1):2721157. doi: 10.1080/16549716.2026.2721157 (PMC13520842; doi:10.1080/16549716.2026.2721157)
Supplement: Supplementary file 2 [file ZGHA_A_2721157_SM5955.docx]

**Supplementary Material 2: Questionnaire**

**Introduction**

I am Samson Udho, a nurse/midwife and currently a student at the University of Cape Town, South Africa where I’m doing research to get a Doctoral Degree. My study is being supervised by Professor Sheila E. Clow who works at the same University. We would like to find out what teenage mothers go through while giving birth in government health facilities. The information you will share may help us to understand what young women in your age group go through while giving birth in government maternity facilities so that we find ways to improve maternity care given to teenagers.

I will read the survey questions to you and give you instructions on how to respond to the questions. Feel free to seek clarification in case you have not heard or understood any of the questions. All your responses will be anonymous & confidential. Also, know that I am not a healthcare worker or a staff in any healthcare facility.

**General information**

a) Has the woman provided written consent to participate in the study?
0. No (*Stop the interview and record as nonresponse*)
1. Yes (*Proceed with the interview*)
b) Questionnaire number (Same as recruitment number): ………………..............
c) Date of childbirth: ……………………………………………………………………
d) Date of interview: ……………………………………………………………………
e) Interviewer’s name: ………………………………………………………………..

**SECTION I: SOCIO-DEMOGRAPHIC CHARACTERISTICS**

Now I am going to ask you some questions about your socioeconomic status and well-being.

1. How old are you in complete years? …………………………..
2. a) Are you currently attending school?
0. No
1. Yes
b) If no, do you plan to return to school following your childbirth?
0. No
1. Yes
3. What is your highest level of education so far?^[1]^
0. No formal education
1. Primary education
2. Secondary education
3. More than secondary education
4. What is your current occupation?^[2]^
0. Agricultural labor
1. Casual labor
2. Salaried worker
3. Self-employed in petty trade
4. Self-employed small-scale industry
5. Unemployed/homemaker
6. Others
5. What is your marital status?
0. Single
1. Married
2. Cohabiting
3. Divorced/separated
4. Widowed
6. What are your estimated household resources (socio-economic index)?

| **S/N** | **Item** | **No=0** | **Yes=1** |
| --- | --- | --- | --- |
| **Durable asset ownership (Tick all that applies)** | | | |
| 1. | Radio |  |  |
| 2. | Television set |  |  |
| 3. | Motor cycle |  |  |
| 4. | Car |  |  |
| 5. | Bicycle |  |  |
| 6. | Telephone |  |  |
| 7. | Fridge |  |  |
| **Source of drinking water supply (Tick one that applies)** | | | |
| 8. | Piped water |  |  |
| 9. | Open well/spring |  |  |
| 10. | Borehole |  |  |
| 11. | River/swamp |  |  |
| **Type of floor material (Tick one that applies)** | | | |
| 12. | Sand/cow dung |  |  |
| 13. | Cement |  |  |
| 14. | Tiles |  |  |
| 15. | Terrazzo |  |  |
| 16. | Others |  |  |

**Section II: Obstetric history**

Now I am going to ask you about your past pregnancy and childbirth.

1. How many times have you been pregnant? ………………………….
2. How many times have you given birth to a baby (that lived or died) that you carried for at least seven months? ………………………………………….
3. How many antenatal clinic visits did you attend during your last pregnancy? ……………………………
4. In which health facility did you give birth?
0. Amach HC IV
1. Ogur HC IV
2. Barr HC III
3. Aromo HC III
4. Agweng HC III
5. Abala HC III
6. Alik HC III
5. a) What was your mode of childbirth for your most recent childbirth?
0. Spontaneous vaginal childbirth
1. Cesarean section
2. Instrumental
3. Don’t know
b) If you had spontaneous vaginal childbirth, did you receive an episiotomy?
0. No
1. Yes
6. What time did you give birth?
0. Day (6:00 am to 2:59 pm)
1. Evening (3:00 pm to 6:59 pm)
2. Night (7:00 pm to 5:59 am)
7. Did you have a birth companion of your choice during your childbirth?
0. No
1. Yes
8. a) Did you have any complications during your childbirth?
0. No
1. Yes
a) If yes, please specify ……………………………………………………..
9. At your most recent childbirth, how many babies did you give birth to?
0. One baby
1. Two babies
2. More than two babies
10. What is (was) the sex of your baby (babies)?
0. Male
1. Female
11. a) Did your baby (babies) have any complications after childbirth?
0. No
1. Yes
b) If yes, please specify ……………………………………………………..
c) Is this baby (babies) still alive?
0. No
1. Yes

**Section III: PCMC during childbirth:**

| “Now I am going to ask you some questions about your experiences in the health facility during your childbirth.  Remember that all the questions in this section refer specifically to the time you were in the health facility for this last childbirth. Also, know that everything you tell me is confidential and will not be shared with the health facility.” | | | | | | | | | | | | | | | | |
| --- | --- | --- | --- | --- | --- | --- | --- | --- | --- | --- | --- | --- | --- | --- | --- | --- |
| **QUESTION** | **RESPONSE OPTIONS** | | | | | | | | | | | | | | | |
| 1. What do you think about the amount of time you waited to be attended to by the doctors, nurses, or other health care providers? Would you say it was very short, somewhat short, somewhat long, or very long? | *Very short* | | | | *Somewhat short* | | | | *Somewhat long* | | | | | *Very long* | | |
|  | 0 | | | | 1 | | | | 2 | | | | | 3 | | |
| 2. During your time in the health facility did the doctors, nurses, or other health care providers introduce themselves to you when they first came to see you? If yes: Was it a few of them, most of them, or all of them? | *No, none of them* | | | | *Yes, a few of them* | | | | *Yes, most of them* | | | | | *Yes, all of them* | | |
|  | 0 | | | | 1 | | | | 2 | | | | | 3 | | |
| “Now I will ask you some questions specifically about how you were treated at the health facility. Tell me if the following things happened all the time, most of the time, a few times, or it never happened. You can say a few times if it happened one or two times, and most of the time will be if it happened 3 or more times, but not always. For some questions, I will ask specifically if something occurred during labor, childbirth, or after childbirth. If I do not specify please answer based on your experiences during the entire time you were in the facility from labor till discharge.”  **Probe for all questions:** If the woman just responds yes, ask them: “Did this occur a few times, most of the time, or all the time?” | | | | | | | | | | | | | | | | |
| 3. Did the doctors, nurses, or other health care providers call you by your name? | *No, never* | | | | *Yes, a few times* | | | | *Yes, most of the time* | | | | | *Yes, all the time* | | |
|  | 0 | | | | 1 | | | | 2 | | | | | 3 | | |
| 4. Did the doctors, nurses, or other staff at the facility treat you with respect? | 0 | | | | 1 | | | | 2 | | | | | 3 | | |
| 5. Did the doctors, nurses, and other staff at the facility treat you in a friendly manner? | 0 | | | | 1 | | | | 2 | | | | | 3 | | |
| 6. During examinations in the labor room, were you covered up with a cloth or blanket or screened with a curtain so that you did not feel exposed? | 0 | | | | 1 | | | | 2 | | | | | 3 | | |
| 7. Do you think your health information was or will be kept confidential at this facility? | 0 | | | | 1 | | | | 2 | | | | | 3 | | |
| 8. Did you feel like the doctors, nurses or other staff at the facility involved you in decisions about your care? | *No, never* | | | *Yes, a few times* | | *Yes, most of the time* | | | | | | *Yes, all the time* | | | *Did not have to make any decisions* | |
|  | 0 | | | 1 | | 2 | | | | | | 3 | | | 4 | |
| 9. Did the doctors, nurses or other staff at the facility ask your permission/ consent before doing procedures on you? | *No, never* | | | | *Yes, a few times* | | | | *Yes, most of the time* | | | | | *Yes, all the time* | | |
|  | 0 | | | | 1 | | | | 2 | | | | | 3 | | |
| 10. During childbirth, were you able to be in the position of your choice? | *No, never* | | | | *Yes, a short time* | | | | *Yes, most of the time* | | | | | *Yes, all the time* | | |
|  | 0 | | | | 1 | | | | 2 | | | | | 3 | | |
| 11. Did the doctors, nurses or other staff at the facility speak to you in a language you could understand? | *No, never* | | | | *Yes, a few times* | | | | *Yes, most of the time* | | | | | *Yes, all the time* | | |
|  | 0 | | | | 1 | | | | 2 | | | | | 3 | | |
| 12. Did the doctors and nurses explain to you why they were doing examinations or procedures on you? | 0 | | | | 1 | | | | 2 | | | | | 3 | | |
| 13. Did the doctors and nurses explain to you why they were giving you any medicine? | *No, never* | *Yes, a few times* | | | | | *Yes, most of the time* | | | | *Yes, all the time* | | | | | *Did not get any medicine* |
|  | 0 | 1 | | | | | 2 | | | | 3 | | | | | 4 |
| 14. Did the doctors and nurses at the facility talk to you about how you were feeling? | No, never | | | | Yes, a few times | | | | | Yes, most of the time | | | Yes, all the time | | | |
|  | 0 | | | | 1 | | | | | 2 | | | 3 | | | |
| 15. Do you think that the doctors, nurses or other staff at the facility tried to understand your anxieties and fears? | *No, never* | *Yes, a few times* | | | | | *Yes, most of the time* | | | | *Yes, all the time* | | | | | *Did not have any anxieties or fears* |
|  | 0 | 1 | | | | | 2 | | | | 3 | | | | | 4 |
| 16. Did you feel you could ask the doctors, nurses or other staff at the facility any questions you had? | No, never | | | | Yes, a few times | | | | Yes, most of the time | | | | | Yes, all the time | | |
|  | 0 | | | | 1 | | | | 2 | | | | | 3 | | |
| 17. Were you allowed to have someone you wanted (from outside of staff at the facility, such as family or friends) to stay with you during labor? | No, never | | Yes, a few times | | | | | Yes, most of the time | | | Yes, all the time | | | | I did not want someone to stay with me | |
|  | 0 | | 1 | | | | | 2 | | | 3 | | | | 4 | |
| 18. Were you allowed to have someone you wanted to stay with you during childbirth? | 0 | | 1 | | | | | 2 | | | 3 | | | | 4 | |
| 19. When you needed help, did you feel the doctors, nurses or other staff at the facility paid attention? | No, never | | | | Yes, a few times | | | | Yes, most of the time | | | | | Yes, all the time | | |
|  | 0 | | | | 1 | | | | 2 | | | | | 3 | | |
| 20. Do you feel the doctors or nurses did everything they could to help control your pain? | 0 | | | | 1 | | | | 2 | | | | | 3 | | |
| 21. Did the doctors, nurses, or other health providers shouted at you, scolded, insulted, threatened, or talked to you rudely? (If yes) will you say this happened once, a few times, or many times? | No, never | | | | Yes, once | | | | Yes, a few times | | | | | Yes, many times | | |
|  | 0 | | | | 1 | | | | 2 | | | | | 3 | | |
| 22. Were you treated roughly like pushed, beaten, slapped, pinched, physically restrained, or gagged? (If yes) will you say this happened once, a few times, or many times? | 0 | | | | 1 | | | | 2 | | | | | 3 | | |
| 23. Did the doctors, nurses, or other staff at the facility ask you or your family for money other than the official cost? | No, never | | | | Yes, a few times | | | | Yes, most of the time | | | | | Yes, all the time | | |
|  | 0 | | | | 1 | | | | 2 | | | | | 3 | | |
| 24. Do you think there was enough health staff in the facility to care for you? | 0 | | | | 1 | | | | 2 | | | | | 3 | | |
| 25. Did you feel the doctors, nurses, or other staff at the facility took the best care of you? | 0 | | | | 1 | | | | 2 | | | | | 3 | | |
| 26. Did you feel you could completely trust the doctors, nurses or other staff at the facility with regards to your care? | 0 | | | | 1 | | | | 2 | | | | | 3 | | |
| **“The next set of questions is about the health facility environment”** | | | | | | | | | | | | | | | | |
| 27. Thinking about the labor and postnatal wards, did you feel the health facility was crowded? | No, never | | | | Yes, once | | | | Yes, a few times | | | | | Yes, many times | | |
|  | 0 | | | | 1 | | | | 2 | | | | | 3 | | |
| 28. Thinking about the wards, washrooms and the general environment of the health facility, will you say the facility was very clean, clean, dirty, or very dirty? | Very dirty | | | | Dirty | | | | Clean | | | | | Very clean | | |
|  | 0 | | | | 1 | | | | 2 | | | | | 3 | | |
| 29. Was there water in the facility? | No, never | | | | Yes, a few times | | | | Yes, most of the time | | | | | Yes, all the time | | |
|  | 0 | | | | 1 | | | | 2 | | | | | 3 | | |
| 30. Was there electricity in the facility? | 0 | | | | 1 | | | | 2 | | | | | 3 | | |
| 31. In general, did you feel safe in the health facility? | 0 | | | | 1 | | | | 2 | | | | | 3 | | |
| **PCMC total Score: 90**  **Dignity and respect full score: 18**  **Communication and autonomy: 27**  **Supportive care: 45** | | | | | | | | | | | | | | | | |

**Section IV: Future Reproductive and Health-seeking Intentions**

Based on your most recent childbirth experience, please choose the number which best indicates your future childbearing intentions.

1. In the future, I would like to have another child:
0. No
1. Yes
2. Don’t know
2. Based on your most recent childbirth experience, where would you choose for your next childbirth?
0. Same health facility
1. Different health facility
2. At my home
3. At somebody else's home
4. Don’t know
3. Do you agree or disagree with this statement: "I would recommend this health facility to a sister or friend"
0. Strongly agree
1. Agree
2. Neutral
3. Disagree
4. Strongly disagree

1. Adapted from Uganda Bureau of Statistics (2017). Note: Education categories refer to the highest level of education attended, whether or not that level was completed (primary education= grade 1 - 7; secondary education = grade 8 - 13).
2. Adopted from Afulani, Sayi, and Montagu (2018)
